# Clinical and epidemiological characteristics of 369 patients with pelvic fractures in Eastern Zhejiang Province of China: a retrospective study

**DOI:** 10.1186/s12891-023-06632-2

**Published:** 2023-06-16

**Authors:** Jiao Dai, Junhong He, Ying Ying, Dichao Huang, Leling Feng

**Affiliations:** 1 Department of Traumatic Orthopedics, Ningbo No. 6 Hospital, Ningbo, China; 2 Department of Pharmacy, Ningbo No. 6 Hospital, Ningbo, China; 3 Department of Nursing, Ningbo No. 6 Hospital, Ningbo, China

**Keywords:** Pelvis, Fracture, falls, Injury, Epidemiological characteristics

## Abstract

**Background:**

Data on the epidemiological characteristics and prognostic factors of patients with pelvic fractures are lacking, particularly in China. This study aimed to summarise the clinical and epidemiological characteristics of patients with pelvic fractures in eastern Zhejiang Province, China, and to identify risk factors for poor prognosis.

**Methods:**

The clinical data of 369 patients with pelvic fractures admitted to the Ningbo No. 6 Hospital between September 2020 and September 2021 were retrospectively analysed. Data on the demographic characteristics; fracture classification; injury time, cause, and site; treatment plan; and prognosis were collected using the Picture Archiving and Communication System and the Hospital Information System. Differences in constituent proportions were analysed using the chi-square test. Logistic regression analysis was used to identify factors affecting patient prognosis. Statistical significance was set at p ≤ 0.05.

**Results:**

Among the 369 patients, there were 206 men and 163 women, at a ratio of 1.26:1, and the average age was 53.64 ± 0.78 years. More than 50% of patients were aged 41–65 years. The average length of hospital stay was 18.88 ± 1.78 days. The three most common causes of pelvic fractures were traffic accidents (51.2%), falls from height (31.44%), and flat-ground falls (14.09%). There were significant differences in the distribution of the three causes of injury depending on age (p < 0.001), sex (p < 0.001), and occupation (p < 0.0001). Most patients were manual workers (48.8%). Furthermore, most patients (n = 262, 71.0%) underwent surgical treatment for pelvic fractures. Postoperative complications occurred in 26 patients (7.05%), and infection was the main complication (73.08%). Age (p = 0.013), occupation (p = 0.034), cause of injury (p = 0.022), treatment options (p = 0.001), and complications (p < 0.0001) were independent factors affecting the prognosis of patients with pelvic fractures. One death (0.027%) occurred, which was due to severe blood loss.

**Conclusions:**

Age, occupation, cause of injury, treatment options and complications were factors affecting patient prognosis. In addition, changes in blood flow and prevention of infection warrant attention.

**Supplementary Information:**

The online version contains supplementary material available at 10.1186/s12891-023-06632-2.

## Background

The pelvis connects and transmits loads between the axial skeleton and lower limbs. As the point of attachment for muscles and ligaments, as well as the space for many blood vessels and nerves, the pelvis has limited movement [1]. Therefore, pelvic fractures are often caused by high-energy trauma, which can be life-threatening to patients without timely treatment [2]. The rate of mortality from pelvic ring fractures is 19%, and this rate increases to 37% in older patients in the presence of severe chest injury or haemodynamic instability [3].

As China is a developing country, the economic development, population, and social environment of its different regions may influence pelvic fracture epidemiology [4]. Currently, there are few reports on the epidemiological characteristics and prognostic factors of patients with pelvic fractures. The Ningbo No. 6 Hospital is an orthopaedic trauma centre in eastern Zhejiang and is the first choice for patients with pelvic fractures in this region. This study aimed to summarise the clinical and epidemiological characteristics of patients with pelvic fractures in the eastern Zhejiang Province, China, and to identify risk factors for prognosis so as to provide a basis for improving the prevention and treatment of pelvic fractures.

## Methods

### Patient data

The clinical data of 369 patients with pelvic fractures admitted to the Ningbo No. 6 Hospital between September 2020 and September 2021 were retrospectively analysed. Data on demographic characteristics; types of pelvic fracture; time, cause, and site of injury; injury site; treatment option; and prognosis were extracted from the Picture Archiving and Communication System and the Hospital Information System.

### Types of pelvic fracture

According to the stability of the pelvis and direction of action of an external force, pelvic fractures can be classified into three types: A, B, or C. Type A (stable type) includes fractures that do not affect the pelvic ring and minor pelvic fractures with complete soft tissue. Type A can be further subdivided into Types A1 (unilateral iliac crest fracture), A2 (unilateral or bilateral pubic branch fracture or iliac fracture), and A3 (transverse tailbone fracture). Type B (partially stable) is rotationally unstable but stable in the vertical direction. It can be further subdivided into Types B1 (anterior injury of the sacroiliac joint), B2 (unilateral compression injury involving ipsilateral posterior iliac ligament injury and anterior pubic and ischial injuries), and B3 (bilateral type B injury). Type C (unstable type) is both rotationally and vertically unstable; it can be further subdivided into Types C1 (digital sacral iliac joint injury), C2 (bilateral sacroiliac joint injury), and C3 (sacral fracture). Acetabular fractures were considered to be different from the aforementioned types and were placed in a separate category. Pelvic fractures that could not be classified into these types were categorized as ‘others’.

### Cause of injury

The causes of injury were divided into the following categories: traffic accidents (injuries caused by vehicles in a state of motion, including collisions between vehicles and collisions between vehicles and people), flat-ground falls (injuries involving a part of the human body touching the ground), falls from height (injuries involving the human body being at a distance from the ground), bruising (injuries caused by objects of mass falling from a height), and crush injuries (injuries caused by objects coming in contact with the human body). Injuries that could not be classified into the abovementioned types were classified as ‘others’.

### Prognosis

In this study, patient prognosis was defined according to the patient’s pain status and activities of daily living 6 months after discharge. During the telephonic follow-up, the pain status was recorded and the Barthel Index was used to assess the activities of daily living. Pain was assessed on the VAS scale, with a score of more than 3 considered significant pain. Patients with no pain and with the ability to perform activities of daily living unassisted were classified as having a good prognosis. Meanwhile, patients with significant pain or who relied on others or tools for activities of daily living were considered to have a poor prognosis.

### Statistical analysis

Statistical analyses were performed using SPSS version 26.0 (IBM Corp). The measurement data had a normal distribution, and descriptive statistics are expressed as mean ± standard deviation and frequencies. Differences in constituent proportions were analysed using the chi-square test. Logistic regression analysis was used to identify factors affecting patient prognosis. Statistical significance was set at p ≤ 0.05. The differences between groups were statistically analysed by post-hoc testing. The absolute value of the adjusted standardised residual in post-hoc testing was limited to 3; when it was greater than 3, we considered the difference between the observed and expected frequency of this value to be statistically significant (Supplementary Tables 2, Additional File 1).

## Results

### Demographic characteristics

**Fig. 1:**
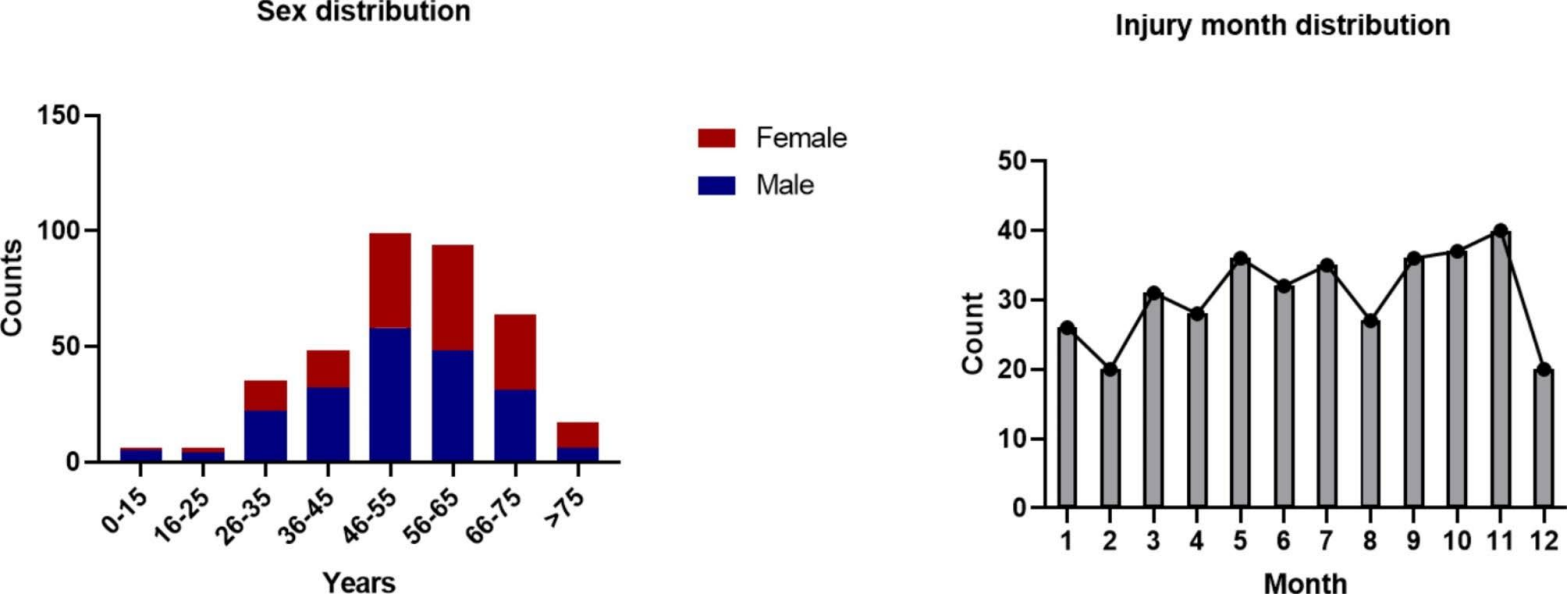
Sex distribution and injury month distribution among patients

Among the 369 patients, there were 206 men and 163 women, at a ratio of 1.26:1, and the average age was 53.6 ± 0.8 years. More than 50% of the patients were aged 46–65 years. In terms of injury duration, the number of patients showed an upward trend from January to November; however, there was a marked decline in December (Fig. 1). Most patients (n = 281; 76.2%) did not have a high school education. Furthermore, most patients were manual workers, followed by unemployed (Table 1).

**Table 1:** Demographic information of patients with pelvic fractures

	Mean	±standard deviation
**Age**	53.6	± 0.8
**Sex**	**Total**	**n (%)**
Male	206	(55.8%)
Female	103	(44.2%)
**Education**	**Total**	**(%)**
Never received education	44	(11.9%)
No high school education	281	(76.2%)
High school diploma or above	44	(11.9%)
**Occupation**	**Total**	**(%)**
Unemployed	105	(28.5%)
Farmer	43	(11.7%)
Manual worker	180	(48.8%)
Office worker	22	(6.0%)
Student	6	(1.6%)
Freelance worker	13	(3.5%)

### Causes of injury

The top three causes of injury were traffic accidents (43.36%), falls from heights (31.44%), and flat-ground falls (14.09%; Supplementary Tables 1, Additional File 1). The top four occupations (95.0%) were selected to analyse the distribution of the causes of injury. Through the chi-square and post-hoc tests, we found that the causes of injury in different occupations significantly differed. The prevalence of falls from heights was higher in manual workers than in those with other occupations, while the prevalence of traffic accidents was lower. Among farmers, the prevalence of flat-ground falls was relatively high compared with that of falls from heights (34.2% versus 13.2%, respectively). Traffic accidents were the main cause of injury in office workers. Flat-ground falls were less common among the unemployed.

Similarly, the data of patients aged 26–65 years were collected for analysis, and we found significant differences in the distribution of causes of injury according to age. Compared with other age groups, in the 66–75-year age group, the prevalence of flat-ground falls increased and that of falls from heights decreased. In addition, falls from heights occurred more frequently in men than in women (Table 2).

**Table 2:** Sex and occupation * cause of injury cross-tabulation

		Cause of Injury			Total	Chi-square test	
		Traffic accidents	Flat-ground falls	Falls from height		χ^2^	p-value
**Sex**						37.460	< 0.001
Male	Count	67	21	89	177		
	(%)	(37.9%)	(11.9%)	(50.3%)	(100.0%)		
Female	Count	93	31	27	151		
	(%)	(61.6%)	(20.5%)	(17.9%)	(100.0%)		
**Total**	Count	160	52	116	328		
	(%)	(48.8%)	(15.9%)	(35.4%)	(100.0%)		
**Age**						31.022	< 0.0001
26–35	Count	13	2	17	32.0		
	(%)	(40.6%)	(6.3%)	(53.1%)	(100.0%)		
36–45	Count	20	3	19	42		
	(%)	(47.6%)	(7.1%)	(45.2%)	(100.0%)		
46–55	Count	35	13	39	87		
	(%)	(40.2%)	(14.9%)	(44.8%)	(100.0%)		
56–65	Count	50	8	25	83		
	(%)	(60.2%)	(9.6%)	(30.1%)	(100.0%)		
66–75	Count	32	18	10	60		
	(%)	(53.3%)	(30.0%)	(16.7%)	100.0%		
**Total**							
	Count	150	44	110	304		
	(%)	(49.3%)	(14.5%)	(36.2%)	(100.0%)		
**Occupation**						58.375	< 0.001
Manual worker	Count	82	18	53	153		
	(%)	(53.6%)	(11.8%)	(34.6%)	(100.0%)		
Unemployed	Count	60	20	19	99		
	(%)	(60.6%)	(20.2%)	(19.2%)	(100.0%)		
Farmer	Count	20	13	5	38		
	(%)	(52.6%)	(34.2%)	(13.2%)	(100.0%)		
Office worker	Count	18	1	2	21		
	(%)	(85.7%)	(4.8%)	(9.5%)	(100.0%)		
**Total**							
	Count	180	52	79	311		
	(%)	(57.9%)	(16.7%)	(25.4%)	(100.0%)		

### Clinical features of patients

The average length of hospital stay was 18.9 ± 1.2 days in the 369 patients, of whom 44 (11.9%) were hospitalised for more than 30 days. Among the 369 pelvic fracture cases, 93 were type A (25.2%), 101 were type B (27.4%), 67 were type C (18.2%), 76 were acetabular fractures (20.6%), and 32 were other types of fractures (8.7%). Eighty-four patients (22.76%) had underlying diseases, including hypertension, diabetes, coronary heart disease, hyperlipidaemia, hyperthyroidism, hepatitis B, depression, asthma, and gout. Thirty-eight patients (10.30%) had more than two underlying diseases. Surgery was the main treatment option among the patients (71.27%); there were 136 cases (36.86%) of open reduction and internal fixation, 71 cases (19.24%) of closed reduction and external fixator fixation, 37 cases (10.03%) of open reduction and internal fixation combined with external fixation, and 19 cases (5.15%) of retrofitting or replacement of joints. Complications occurred in 21 patients (5.69%) and included infection (n = 19; 90.48%), diarrhoea (n = 1; 4.76%), intestinal obstruction (n = 1; 4.76%), respiratory failure (n = 2; 9.52%), gastrointestinal bleeding (n = 1; 4.76%), and allergies (n = 2; 9.52%). After treatment in our hospital, 342 patients (92.68%) had a good prognosis and 27 patients (7.32%) had a poor prognosis, including one death due to severe blood loss (Supplementary Tables 3, Additional File 1).

### Regression analysis

Logistic regression analysis showed that age (p = 0.013), occupation (p = 0.034), cause of injury (p = 0.022), treatment options (p = 0.001), and complications (p < 0.0001) were independent factors affecting the prognosis of patients with pelvic fractures (Table 3).

**Table 3:** Logistic regression analysis of factors affecting patient prognosis

Index	Wald	Df	p-value
Age	6.014	1	0.013*
Occupation (classified)	12.068	5	0.034*
Education (classified)	0.595	2	0.743
Cause of injury (classified)	13.148	5	0.022*
Type of pelvic fracture (classified)	2.583	4	0.630
Complications (classified)	27.305	1	< 0.0001*
Underlying disease (classified)	2.602		0.107
Treatment option (classified)	11.666	1	0.001*

* indicates that the data were statistically significant

## Discussion

Owing to high-energy impact, pelvic fractures are often accompanied by multiple fractures and organ injuries [5]. At the same time, because the pelvic ring contains critical blood vessels of the lower extremities, trauma and fracture can cause haemodynamic changes, significantly increasing the risk of death [6–8]. In the China National Fracture Study, more than 400 cases of pelvic fractures occurred among 512,187 Chinese residents between 2014 and 2015, accounting for 0.09% of cases [9]. The incidence of pelvic fractures determines the need for epidemiological studies to collect data on a sufficient number of cases for long-term analysis. As the cause and treatment of pelvic fractures are often related to socioeconomic development and medical infrastructure [10], errors have been introduced during data collection over long periods. Retrospective analysis of large samples over a short period has rarely been reported.

In contrast to the findings of the study conducted by Li et al. [5], we found that the occurrence of pelvic fractures did not have a consistent trend but showed a fluctuating upward trend. However, more significant declines were observed in February, August, and December. The Spring Festival, the most important traditional Chinese festival, occurred in mid-February. August is the warmest month during summer in China, while December is the coldest, with most enterprises entering a resting period. All these factors could lead to a decline in the frequency of people going out to work and partaking in activities. We speculate that these reasons are why the number of patients with pelvic fractures appears to fall during these periods [11, 12]. The peak age of sustaining pelvic fractures ranged between 46 and 65 years. The male-to-female ratio was 1.26:1, which is smaller than the ratio of 1.5:2.5 found in previous studies [13–16]. This suggests that opportunities for women to participate in high-intensity, high-risk work and social activities are increasing.

As in previous studies, the top three causes of injury were traffic accidents, falls from heights, and flat-ground falls [9, 17]. Notably, in this study, one in five traffic accidents involved patients who rode electric bicycles. With the rapid development of China’s economy and the improvement in road conditions, the number of private cars in the country has increased. Traffic management of private cars is constantly being strengthened in China [16]. However, control measures for electric bicycles still need to be improved. Although the prevalence of falls from heights was less than that of traffic accidents, falls from heights have the highest risk of affecting patient prognosis. Most patients who fell from heights were manual workers, and work demands and fatigue easily induced the occurrence of falls from heights. The high number of traffic accidents may be attributed to a low level of education among many Chinese residents, which leads to a lack of safety awareness and the inability to understand and obey traffic rules. In addition, a low education level may limit these residents to outdoor work or labour, which does not require an advanced degree.

External fixation can reconstruct the pelvic ring and has great advantages in restoring pelvic volume and controlling bleeding; however, its effect on maintaining fracture reduction is inferior to that of internal fixation [18]. Therefore, internal fixation with a short operation time and maximum pelvic fixation is the main choice for surgery [19]. In addition, internal fixation can be combined with external fixation. Underlying diseases did not appear to affect the final prognosis of the patients in this study. Complications were the most important risk factors for poor prognosis; among the 21 patients with complications, 18 had infections, including 9 cases of pulmonary infection. The effect of blood loss on prognosis was not statistically significant. However, the only death that occurred in this study was due to severe blood loss. This is a reminder that close attention should be paid to changes in blood flow, and the prevention of infection warrants attention in clinical practice.

The limitations of this study include the small number of patients with a poor prognosis and the lack of further classification of influencing factors, such as specific surgical procedures and the degree of poor prognosis. In addition, as only one death occurred, we were unable to assess survival or the causes of death. Furthermore, as we only analysed data collected over 1 year, we were unable to evaluate the potential influence of other factors, such as the changes in the information of patients with pelvic fractures from year to year.

## Conclusions

Age, occupation, cause of injury, treatment options and complications were factors affecting patient prognosis. Traffic accidents were the most common cause of injury, while fall injuries posed the greatest threat to patient health. Thus, education on traffic rules and safety awareness must be strengthened. Surgical treatment should be performed as far as possible. In addition, changes in blood flow and prevention of infection warrant attention.

## Electronic supplementary material

Below is the link to the electronic supplementary material.

Supplementary Material 1

## Data Availability

All data generated or analysed during this study are included in this published article and its supplementary information files.
