# Supplementary material for: Clinical and epidemiological characteristics of 369 patients with pelvic fractures in Eastern Zhejiang Province of China: a retrospective study
Source: BMC Musculoskelet Disord. 2023 Jun 16;24:495. doi: 10.1186/s12891-023-06632-2 (PMC10273619; doi:10.1186/s12891-023-06632-2)
Supplement: Supplementary file 1 — Supplementary Material 1 [file 12891_2023_6632_MOESM1_ESM.docx]

Additional File 1

Supplementary Table 1. Frequency of causes of injury

| **Cause of injury** | **Total** | **n (%)** |
| --- | --- | --- |
| Traffic accident | 160 | (43.36%) |
| Flat ground fall | 52 | (14.09%) |
| Fall from a height | 116 | (31.44%) |
| Bruise | 16 | (4.34%) |
| Crush | 14 | (3.79%) |
| Other | 11 | (2.98%) |

| Supplementary Table 2. Cross-tabulation of adjusted residuals | | | |
| --- | --- | --- | --- |
|  | **Traffic accidents** | **Flat ground falls** | **Falls from heights** |
| **Sex** |  |  |  |
| Male | -4.3 | -2.1 | 6.1 |
| Female | 4.3 | 2.1 | -6.1 |
| Age |  |  |  |
| 26–35 | -1.0 | -1.4 | 2.1 |
| 36–45 | -0.2 | -1.5 | 1.3 |
| 46–55 | -2.0 | 0.1 | 2.0 |
| 56–65 | 2.3 | -1.5 | -1.3 |
| 66–75 | 0.7 | 3.8 | -3.5 |
| **Occupation** |  |  |  |
| Manual worker | -4.8 | 2.3 | 6.9 |
| Unemployed | 2.9 | 1.1 | -3.9 |
| Farmer | 0.5 | 3.1 | -3.0 |
| Office worker | 3.5 | 1.5 | -2.5 |

Supplementary Table 3. Clinical features of patients with pelvic fractures

|  | [**Mean**](javascript:;) | | **±SD** |
| --- | --- | --- | --- |
| **Length of hospital stay**  **(days)** | 18.9 | | ±1.2 |
|  | | **Total** | **n (%)** |
| **Treatment option** | |  |  |
| Surgery | | 106 | (28.7%) |
| Conservative treatment | | 263 | (71.3%) |
| [**Prognosis**](javascript:;) | |  |  |
| Good | | 342 | (92.7%) |
| Poor | | 26 | (7.1%) |
| Death | | 1 | (0.3%) |
| **Types of pelvic fracture** | |  |  |
| Type A | | 93 | (25.2%) |
| Type B | | 101 | (27.4%) |
| Type C | | 67 | (18.2%) |
| Acetabular fracture | | 76 | (20.6%) |
| Other | | 32 | (8.7%) |
| [**Underlying disease**](javascript:;) | |  |  |
| None | | 274 | (66.9%) |
| One | | 84 | (22.7%) |
| Two | | 28 | (7.6%) |
| - Three or more | | 10 | (2.7%) |
| **Surgical method** | |  |  |
| Open reduction and internal fixation | | 136 | (36.9%) |
| Closed reduction and external fixator fixation | | 71 | (19.2%) |
| Open reduction and internal fixation combined with external fixation | | 37 | (10.0%) |
| Retrofitting or replacement of joints | | 19 | (5.2%) |
| [**Complication**](javascript:;)**s** | | Total |  |
| Infection | | 19 |  |
| Diarrhoea | | 1 |  |
| Intestinal obstruction | | 1 |  |
| Respiratory failure | | 2 |  |
| Gastrointestinal bleeding | | 1 |  |
| Allergy | | 2 |  |

SD, standard deviation
